# Fast and convenient delivery of fluidextracts liquorice through electrospun core-shell nanohybrids

**DOI:** 10.3389/fbioe.2023.1172133

**Published:** 2023-04-06

**Authors:** Hang Liu, Yelin Dai, Jia Li, Ping Liu, Wenhui Zhou, Deng-Guang Yu, Ruiliang Ge

**Affiliations:** ^1^ School of Materials and Chemistry, University of Shanghai for Science and Technology, Shanghai, China; ^2^ Wenqi Middle School, Shanghai, China; ^3^ Qingpu Campus, High School Affiliated to Fudan University, Shanghai, China; ^4^ Institute of Infectious Disease and Biosecurity, School of Public Health, Fudan University, Shanghai, China; ^5^ The Base of Achievement Transformation, Shidong Hospital Affiliated to University of Shanghai for Science and Technology, Shanghai, China; ^6^ Institute of Orthopaedic Basic and Clinical Transformation, University of Shanghai for Science and Technology, Shanghai, China; ^7^ Department of Outpatient, The Third Affiliated Hospital, Naval Medical University, Shanghai, China

**Keywords:** coaxial electrospinning, core–shell structure, liquorice, solidification, dosage forms, process–structure–performance relationship

## Abstract

**Introduction:**

As an interdisciplinary field, drug delivery relies on the developments of modern science and technology. Correspondingly, how to upgrade the traditional dosage forms for a more efficacious, safer, and convenient drug delivery poses a continuous challenge to researchers.

**Methods, results and discussion:**

In this study, a proof-of-concept demonstration was conducted to convert a popular traditional liquid dosage form (a commercial oral compound solution prepared from an intermediate licorice fluidextract) into a solid dosage form. The oral commercial solution was successfully encapsulated into the core–shell nanohybrids, and the ethanol in the oral solution was removed. The SEM and TEM evaluations showed that the prepared nanofibers had linear morphologies without any discerned spindles or beads and an obvious core–shell nanostructure. The FTIR and XRD results verified that the active ingredients in the commercial solution were compatible with the polymeric matrices and were presented in the core section in an amorphous state. Three different types of methods were developed, and the fast dissolution of the electrospun core–shell nanofibers was verified.

**Conclusion:**

Coaxial electrospinning can act as a nano pharmaceutical technique to upgrade the traditional oral solution into fast-dissolving solid drug delivery films to retain the advantages of the liquid dosage forms and the solid dosage forms.

## 1 Introduction

As an interdisciplinary field, drug delivery sources information mainly from three areas, the knowledge of pharmacokinetics and the pharmacology of drugs *in vivo* (Niu et al., 2021a; Wang et al., 2022; Meng et al., 2022; Tang et al., 2022; Wang and Feng, 2022; Wu et al., 2022), new types of pharmaceutical excipients for carrying the drug molecules to the lesion locations (Murugesan and Raman, 2021; Li C. F. et al., 2022; Wang L. et al., 2022; Zhu et al., 2022; Wang et al., 2023; Kostka et al., 2023), and new pharmaceutical techniques and related strategies, which are often introduced from other disciplines (Chen et al., 2022; Xie et al., 2022; Song et al., 2023a; Wang et al., 2023; Chen et al., 2023; Huang and Feng, 2023). During the past several decades, the effective combination of these three aspects has continuously updated the commercial drug dosage forms for a more efficient, safer, and more convenient drug administration process (Fatema et al., 2022; Hopkins et al., 2022; Shen et al., 2022; Zhao et al., 2023). In other word, upgrading drug dosage forms comprises one of the most important challenges in the pharmaceutical sciences both on an industrial scale and in laboratory investigations.

**FIGURE 1:**
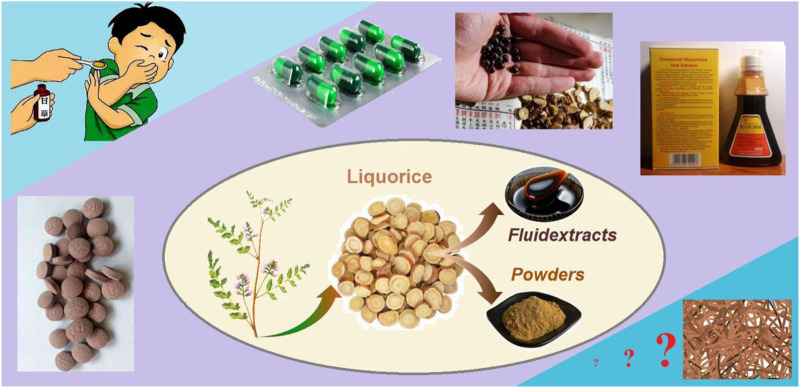
The processes from the licorice plant to the intermediate dosage forms of licorice fluidextracts and powders and to several types of commercial dosage forms, including tablet and buccal tablet, capsule, drop pill, and compound oral solution.

Licorice (also termed as radix liquiritiae or liquiritia glycyrrhiza) is one of the most popular herbs (Ding et al., 2022; Yan et al., 2022). It contains several types of phytochemical active ingredients, which are useful for treating a series of diseases such as cough, sputum, inflammation, cancer, diabetes, and also COVID-19 (Xie et al., 2014; Wang et al., 2020; Zhang et al., 2021). It has been developed into many kinds of dosage forms for oral administration because of its effectiveness in treating cough and sputum in Chinese medicinal markets. Shown in Figure 1 are the common processes from the licorice plant (mainly roots and stems) to the intermediate dosage forms of licorice fluidextracts and powders. Later, they can be converted into the commercial dosage forms, including tablet and buccal tablet, capsule, drop pill, and compound oral solution. Among these, the compound oral solution is popular with patients due to its effectiveness as an antitussive and expectorant and its simple administration. However, there are several inevitable disadvantages that have limited its applications, particularly for children. These disadvantages include a certain content of ethanol (which is left during the extraction process), a special oral administration procedure (a small vessel is needed to measure a certain volume compound solution, often 5–10 mL for each time), as well as the stability issues associated with the liquid dosage forms.

Today, electrospinning, as a useful nanofiber fabrication method, has spread into almost all scientific applied fields (Pang et al., 2021; Li et al., 2022; Wang et al., 2023; Wang et al., 2023; Geesi et al., 2023), including several biomedical applications, such as tissue engineering, regeneration medicine, and wound dressing (Khazaeli et al., 2020; Niu et al., 2021b; Li et al., 2021; Maleki et al., 2021; Zhao H. et al., 2022; Gao et al., 2022; Liang et al., 2022). Particularly in pharmaceutics, it is regarded as a popular pharmaceutical nanotechnology (Spizzirri et al., 2021; Chen et al., 2022; Wang et al., 2022). As the most attractive electrohydrodynamic atomization (EHDA) process, it is more applicable than other EHDA processes such as electrospraying and e-jet printing (Li et al., 2023; Li et al., 2023). In the literature, electrospun drug-loaded nanofibers have been extensively investigated for their potential to resolve some key issues in pharmaceutics, such as the dissolution of poorly water-soluble drugs; the oral delivery of peptides, proteins, and vaccines; anti-drug resistance, and the effective crossing of the major human body barriers (the blood–brain barrier, the placental barrier, and the skin) for efficacious therapy (Vass et al., 2020; He et al., 2021; Köse et al., 2021; Lv et al., 2021; Jiang et al., 2022a; Pattnaik et al., 2022; Yu and Zhao, 2022; Yu, 2023). Electrospinning can allow for the possibility of new licorice commercial products. In particular, electrospinning is developing very quickly, expanding its capability of creating new nanofibers and providing powerful tools for constructing novel drug delivery systems (DDSs). These directions include: 1) production on an industrial scale to create “more” nanofibers (Zhao et al., 2022; Cao et al., 2022; Cui et al., 2023); 2) creating complicated nanostructures to load more kinds of active ingredients to endow functionally “better” nanofibers (Keirouz et al., 2020; Huang et al., 2022; Li et al., 2022; Wang et al., 2023e); and 3) combination with traditional physical and chemical methods to convert more materials into nanofibers with “stronger” commercial competitiveness (Huang et al., 2022; Huang et al., 2022; He et al., 2022; Xu et al., 2023).

Based on the abovementioned knowledge, we hypothesize that a coaxial electrospinning process can be developed to convert the unspinnable commercial compound licorice oral solutions (prepared from the intermediate licorice fluidextracts) into solid nanofibers. The active ingredients from licorice can be sealed into the core sections of structural nanofibers with a polyvinylpyrrolidone (PVP) shell protection, which is a frequently used polymer (Kang et al., 2020; Brimo et al., 2023; Wei et al., 2023). Meanwhile, sucralose can be loaded into the sheath section to endow the nanofibers with a favorable taste for simple oral administration. The implementation of several EHDA processes was carefully pretested. The resultant core–shell nanofibers were assessed in terms of their morphologies, inner structures, compatibility, and physical state. Three different types of methods were developed to evaluate the fast dissolution of the core–shell nanofibers.

## 2 Materials and methods

### 2.1 Materials

Polyvinylpyrrolidone K90 (*M*_w_ = 1,300,000) and K30 (*M*_w_ = 58,000) were purchased from Sigma-Aldrich Corp (Shanghai, China). Analytical grade ethanol was obtained from the No. 3 Reagent Factory (Shanghai, China). Compound Glycyrrhiza Oral Solution (Chinese medicine approval: H31020828) was purchased from Shanghai Laobaixing Big Pharmacy, which was fabricated by Shanghai Mei You Pharmaceutical Co., Ltd. (Shanghai, China). The production date, product batch number, and expiry date were 2022-06-23, 220606, and 2025-06-22, respectively. Water was double distilled just before usage.

### 2.2 Three types of EHDA processes

The homemade apparatus for conducting the EHDA processes comprised four parts, i.e., two syringe pumps for quantitatively driving the core and shell working fluids (KDS100 and KDS 200, Cole-Parmer, Vernon Hills, lL, United States), a power supply (ZGF/60kV, Wuhan Huatian Co., Ltd. Wuhan, China), a collector formed by wrapping aluminum foil around cardboard, and a homemade concentric spinneret.

First, 50 mL of the commercial oral solution was measured in triplicate. Weights of 0.0, 5.0, and 10.0 g of PVP K30 were added into the solutions, respectively. The electrospraying processes used these three brown solutions to prepare the S1, S2, and S3 samples, respectively (Table 1). The shell electrospinnable fluid was prepared by co-dissolving 2.0 g sucralose and 8.0 g PVP k90 in 100 mL ethanol with a single-fluid electrospinning through the shell passage of the concentric spinneret to fabricate the S4 sample.

**TABLE 1:** The experimental parameters for conducting the three kinds of EHDA processes.

Sample	EHDA process	Applied voltage (kV)	Pumping rate (mL/h)		Product
			Sheath^a^	Core^b^	
S1	Electrospraying	8	0.0	0.5	Liquids
S2		12	0.0	0.5	Liquids
S3		14	0.0	0.5	Liquids
S4	Electrospinning	10	2.0	0.0	Nanofibers
S5	Coaxial	15	2.0	0.5	Core–sheath nanofibers
S6	electrospinning	15	2.0	1.0	Hybrids

^a^ A ratio of 2% (w/v) sucralose and 10% (w/v) PVP K90 were dissolved into ethanol.

^b^ Amounts of 0%, 10%, and 20% PVP, K30 were dissolved into 50 mL commercial solution to prepare S1, S2, and S3, respectively. The third solution was explored as the core fluid to prepare S5 and S6.

Two kinds of core–shell nanofibers, i.e., the S5 and S6 samples were prepared using the coaxial electrospinning processes. The shell fluid flow rate was kept at 2.0 mL/h, and the core fluid flow rates were adjusted to 0.5 and 1.0 mL/h for S5 and S6, respectively. The distance between the tip of the spinneret and the collector was kept at a fixed value of 20 cm. The applied voltages were adjusted under the criterion that the EHDA processes would be continuously and robustly carried out, and the values are included in Table 1.

### 2.3 Characterizations of the physical properties

#### 2.3.1 Morphology

The morphologies of the solid EHDA products including samples of the S4 monoaxial nanofibers and the S5 and S6 core–shell nanofibers were assessed using a field-emission scanning electron microscope (SEM, Quanta FEG450, Hillsboro, United States). The SEM pictures were used to estimate the average diameters of the nanofibers in about 100 places using the ImageJ software (National Institutes of Health, Bethesda, United States). The sampling processes included cutting a small piece of fibrous mat, which was fixed on a sample table using a double-sided conductive adhesive, and a thin layer of Pt was sprayed for 60 s before the sample was placed into the vacuum chamber for assessment.

#### 2.3.2 Inner structure

The inner structures of the S4 monolithic nanofibers and the S5 core–shell nanofibers were evaluated using a transmission electron microscope (TEM, JEM2200F, JEOL, Japan). The samples were prepared by placing a carbon film supported by a 200 mesh copper mesh around the fiber collector for about 2 min; then, the collected nanofibers were put into the vacuum chamber for detection.

#### 2.3.3 Physical state of components

X-ray diffraction (XRD) tests were carried out using the Bruker X-ray Powder diffractometer (Bruker-AXS, Karlsruhe, Germany). The raw materials and their fiber mats were measured within a 2θ angle range of 5°–60°. The applied voltage and working current were 40 kV and 30 mA, respectively. The scanning 2θ range was from 10° to 60° with a rotation speed of 5° per minute).

#### 2.3.4 Compatibility

Fourier transform infrared (FTIR) analyses were implemented using the PerkinElmer FTIR Spectrometer (Spectrum 100, Billerica, United States). The experiments were performed in range 500–4,000 cm^−1^ with a resolution of 2 cm^−1^. The sampling for the solid materials included weighing 0.2 g of potassium bromide powder, grinding it with about 10 mg of the sample, pressing the mixture into solid tablets, and placing the tablets into the instrument for scanning. As for the commercial oral solution, a drop of its liquid was dripped onto a blank potassium bromide tablet for measurement.

### 2.4 Functional performance assessments

New methods for human health are always highly desired. In this study (Bai et al., 2021; Li C. Q. et al., 2023), three methods were developed for assessing the fast dissolution performances of the S5 electrospun core–shell nanofibers. A glass slide was placed above the collector to collect the S5 core–shell nanofibers for about 2 h. Later, two experiments were conducted. One was to place a drop of water on the collected fibrous film, and the dissolution processes were recorded by a camera (PowerShot A490, Canon, Tokyo, Japan). The other was a drop shaped analysis instrument (DSA100, Kruss GmbH, Hamburg, Germany), which was used to determine the surface water contact angle with a small volume of 3 μL double-distilled water. The processes of the water droplet were recorded by the instrument.

The third experiment was an artificial tongue experiment. A piece of paper was wetted on the laboratory desk as an artificial tongue. A piece of the collected core–shell fibrous mat S5 was cut and placed on the surface of the wet paper. The process of the film’s disappearance was recorded using a digital camera.

## 3 Results and discussion

### 3.1 The implementation of the different types of EHDA processes

**FIGURE 2:**
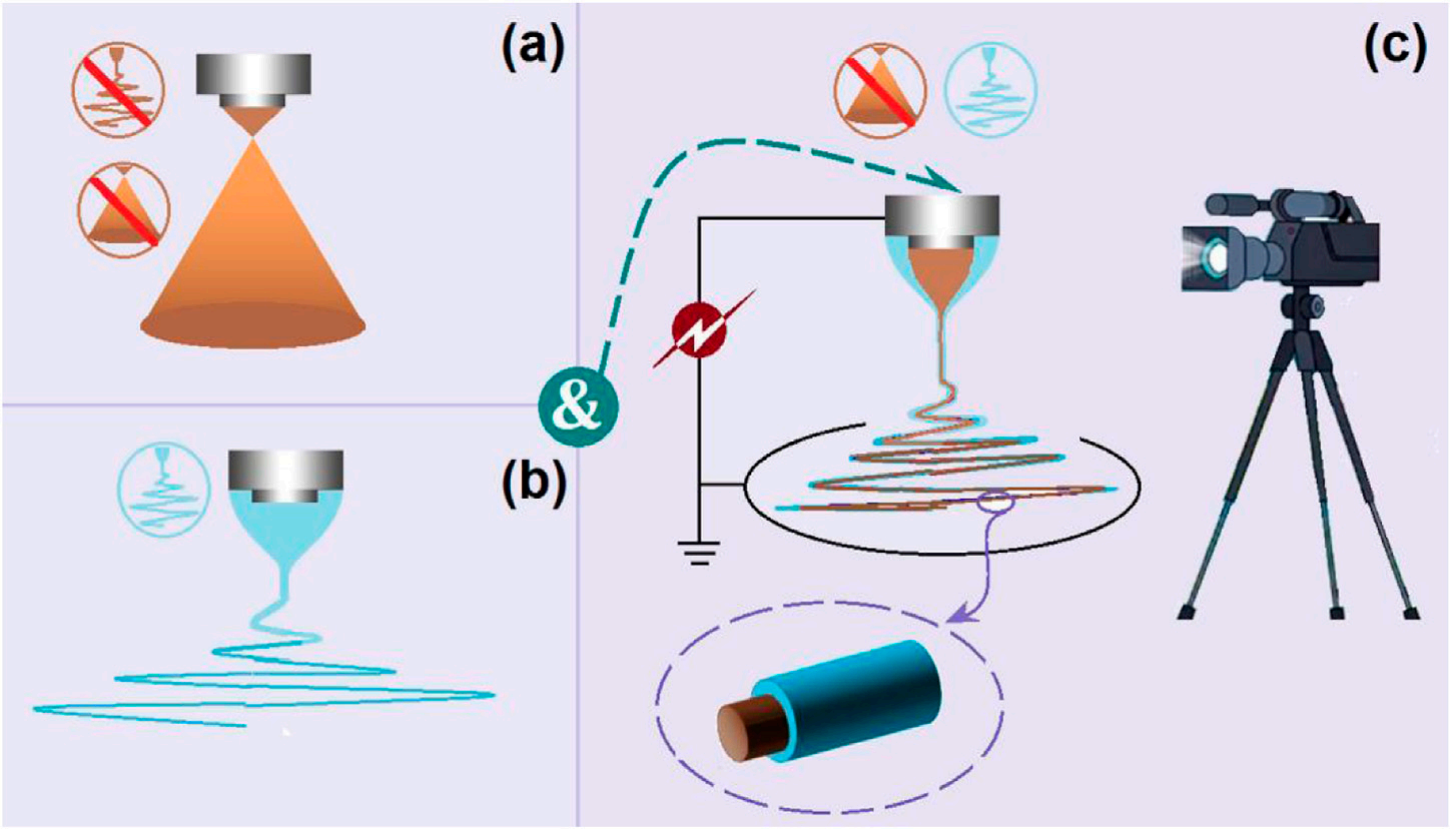
A combination of electrospraying and single-fluid blending electrospinning to realize a modified coaxial electrospinning process: **(A)** electrospraying; **(B)** single-fluid electrospinning; **(C)** the modified coaxial electrospinning and the resultant core–shell nanofibers.

The EHDA processes mainly take advantage of the quick and simple interactions between the electrostatic energy and working fluids, by which the solvents can be repelled (Abadi et al., 2022; Sivan et al., 2022a; Chen et al., 2022; Han et al., 2022; Yao et al., 2023). Thus, to prepare solid products, first and foremost the solvents must be removed all at once during the working processes. Correspondingly, the success or failure of an electrospinning process is often evaluated by the electrospinnability of the working fluid (Sivan et al., 2022b; Huang et al., 2022; Krysiak and Stachewicz, 2022; Lu et al., 2023). The traditional single-fluid electrospinning is limited by a series of subjective elements, such as the filament-forming property of polymers, the narrow spinnable window of polymers with a filament-forming property, suitable solvents for preparing the working fluids, and the co-dissolution of the polymer and the active ingredients in one solvent (or a solvent mixture) (Xu et al., 2023; Yao et al., 2023; Tabakoglu et al., 2023). Only one of the working fluids must be electrospinnable when the treating working fluid number is larger than two. This case has greatly expanded the capability of electrospinning in treating more kinds of liquids and correspondingly enabled the creations of new types of complex nanostructures through intentionally tailoring the locations of the components within the nanofibers (Zhou et al., 2022; Abdul Hameed et al., 2023). In this study, the commercial compound licorice solution had no electrospinnability and could not be solidified using an electrospraying process. Thus, a combination strategy of electrospraying and single-fluid blending electrospinning was developed to convert the solution into a solid product (Figure 2). Shown in Figures 2A, B are diagrams of a typical electrospraying and electrospinning process, respectively. When the two fluids were simultaneously driven to the concentric spinneret, a coaxial electrospinning process was conducted; provided the operation conditions were correct, the core–shell nanostructure was ensured (Figure 2C).

**FIGURE 3:**
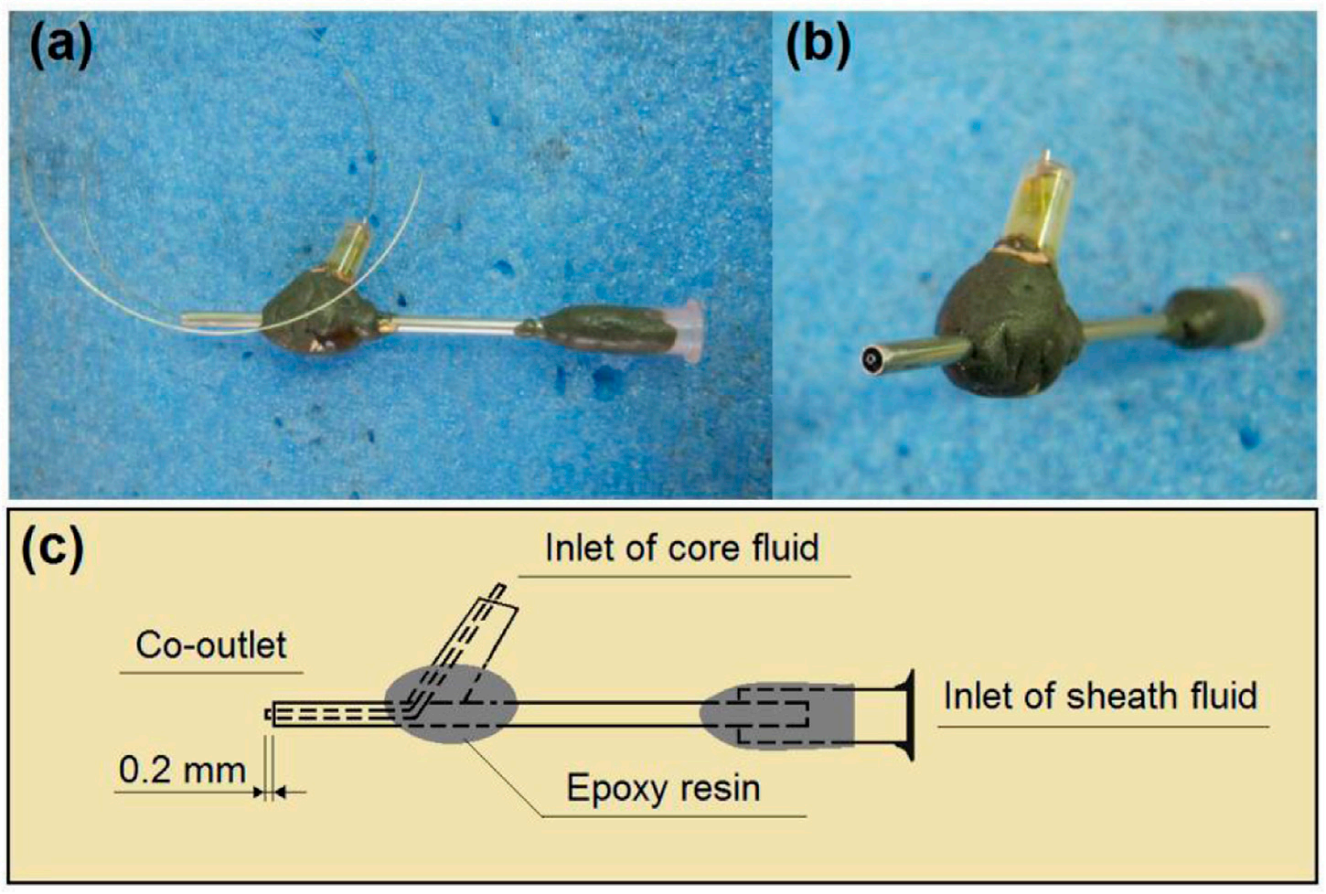
Details of the homemade concentric spinneret: **(A)** the spinneret; **(B)** the co-outlet of the spinneret’s nozzle; **(C)** the inner connections of the capillaries.

In an electrospinning system, there are typically four parts, i.e., a power supply for furnishing the high voltage, a collector for the nanofiber deposition, a spinneret to guide the fluid (or several fluids simultaneously), and a pump (for a single-fluid blending electrospinning process or several pumps for driving several working fluids to the spinneret’s nozzle in a quantitative manner) (Jiang et al., 2022b; Lv et al., 2022; Li et al., 2023). Among them, the spinneret is the most important part, because it determines the electrospinning types and the structures of the resulting nanofibers (Bai et al., 2022; Song et al., 2023b; Lv et al., 2023). In this study, the details of the homemade concentric spinneret are shown in Figure 3. Figure 3A is a digital photo of the spinneret; the inner diameter of the core stainless steel capillary was 0.1 mm. A stainless steel wire with a diameter of 0.09 mm was utilized to clean the spinneret after finishing an electrospun production. Figure 3B is a view of the co-outlet of the spinneret’s nozzle, the sheath, and the core capillaries presented in a concentric manner. These two capillaries had a common axial, thus a coaxial electrospinning process. Figure 3C provides information about the inner connections of the capillaries and the fixing with epoxy resin.

**FIGURE 4:**
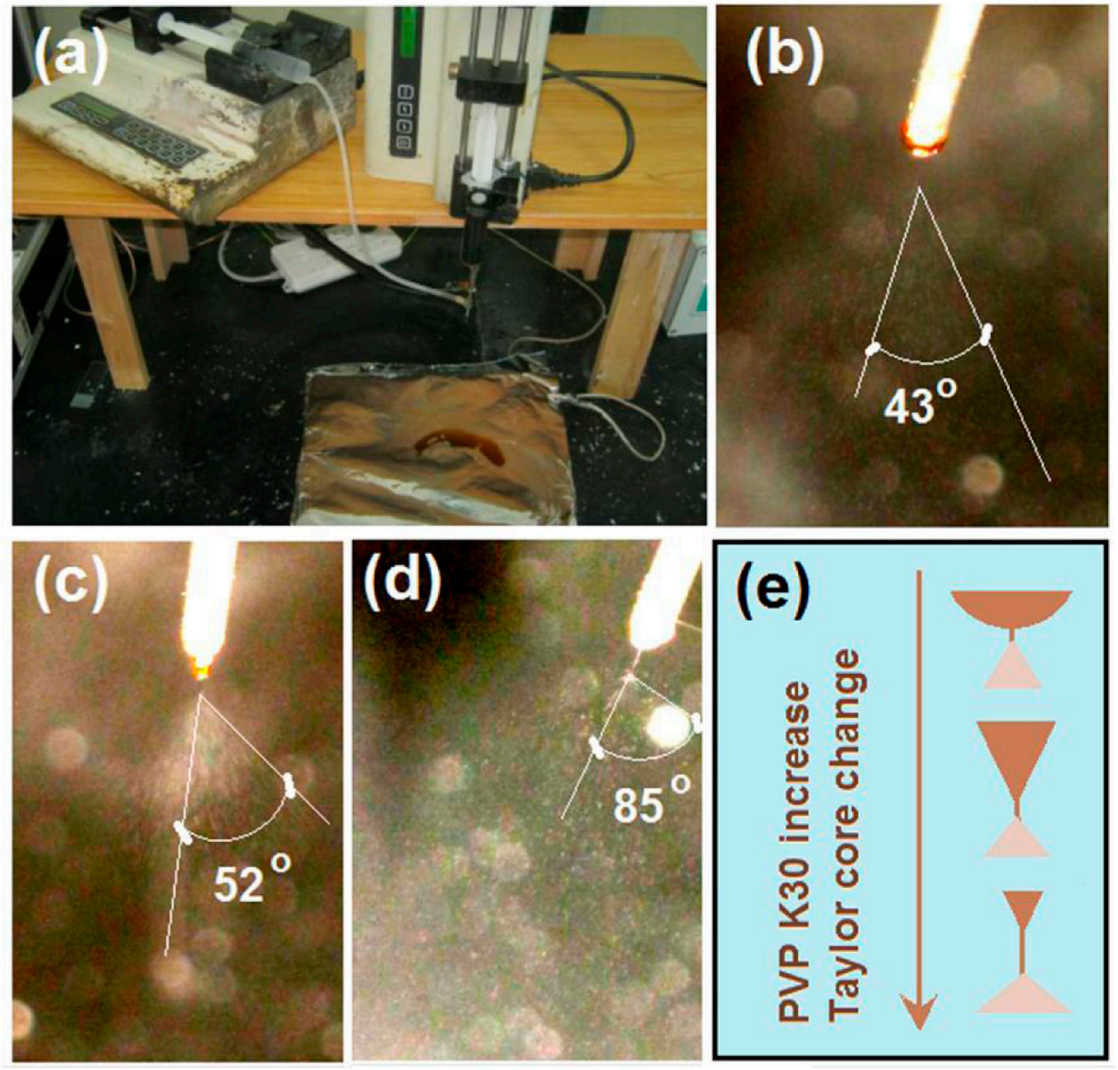
The electrospraying processes with the inner capillary of the concentric spinneret as the fluid guided tube: **(A)** the working system; **(B)** the treatment process of S1; **(C)** the treatment process of S2; **(D)** the treatment process of S3; **(E)** the changes in the Taylor cone with the increase in the PVP K30 in the commercial oral licorice solution from 0% to 10% (w/v) to 20% (w/v), respectively.

In the multiple-fluid processes, one major issue is the compatibility between the different working fluids (Wang et al., 2020; Liu et al., 2022; Wang et al., 2023f; 2023g). The solutes cannot coagulate in another fluid, and their properties should be matched in a certain range, particularly their viscosities. In this study, the commercial compound licorice solution was very dilute. To increase its viscosity to match the electrospinnable shell PVP/sucralose solution for a simple coaxial process and to ensure the fast dissolution of the core–shell nanofibers, PVP K30 was added as a thickening agent. The S1 to S3 samples were the products of a blended solution of commercial compound licorice solution with a certain amount of PVP K30. Their electrospraying processes are recorded in Figure 4. During these processes, the shell fluids were switched off to let only the core fluid pass through the nozzle of spinneret. Figure 4A is a digital photo of the preparation of the S3 sample. Figures 4B–D are the recorded phenomena of the spinneret’s nozzle while preparing the S1, S2, and S3 samples, respectively. In Figure 4A, it is clear that the working fluid did not solidify for S3, as with the reports in the literature, although it had a high concentration of 20% (w/v) PVP K30, as indicated by the collected brown liquid on the collector. This had a close relationship with the solvent of the commercial solution (mainly water and with a small portion of ethanol from the raw licorice fluidextract). In the literature and also in industry, the raw licorice fluidextract is often dried using a heat spraying technique to make solid powders (Lu et al., 2013; Taarji et al., 2021), which may have negative influences on the extracted active ingredients. Similarly, the pure commercial compound solution with an addition of 10% (w/v) for preparing the S1 and S2 samples did not result in solid particles. Non-etheless, the changes in the Taylor cone and the *in situ* spraying processes are interesting. The change trend is sketched in Figure 4E. When the contents of the PVP K30 in the core fluids increased from 0% (w/v) to 20% (w/v), the viscosities and surface tensions correspondingly increased. Thus, a higher applied voltage was needed to initiate the electrospraying, whose values were 8.2, 11.4, and 12.6 kV for preparing S1 to S3, respectively. Under these conditions, the Taylor cones changed their outer shapes from a circular arc to a “fat triangle” and to a “slim triangle”. Meanwhile, the spread angles of the atomization region were enlarged from 43o to 52o and 85o, respectively. In addition, during the spraying process, the Taylor cones were shown to hang on the whole nozzle of the spinneret although they were pumped from the core capillary. The reason could be the combined effect of the capillary siphon phenomenon from the shell passages and the simple infiltration of the core aqueous solutions onto the stainless steel surface. A polymer-based spinneret may have a better result in rejecting the spread of the working fluid.

**FIGURE 5:**
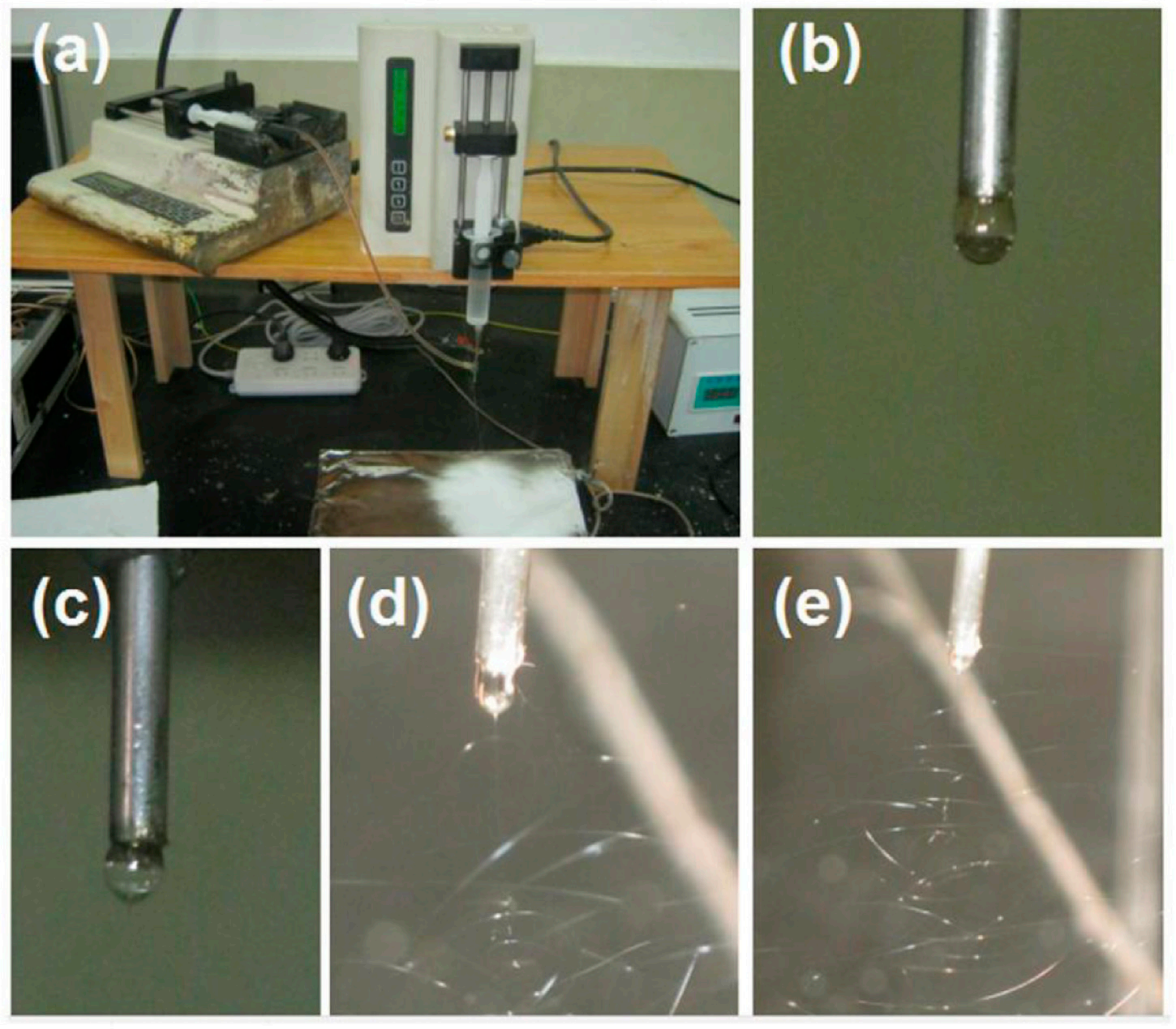
The single-fluid blending process with the outer capillary of the concentric spinneret as the fluid guided tube: **(A)** the spinning apparatus; **(B)** the natural formation of a droplet from the PVP-sucralose solution; **(C)** the change in the droplet shape when an applied voltage of 2 kV was provided; **(D)** the electrospinning process under an applied voltage of 6 kV; **(E)** the electrospinning process for preparing the S4 nanofibers under an applied voltage of 12 kV.

When the core fluid stopped pumping to the spinneret’s nozzle, a traditional single-fluid blending electrospinning of the PVP/sucralose solution was conducted to produce the S4 sample. The whole process is shown in Figure 5A, with white fibrous mats depositing on the collector. During the electrospinning, the operation procedure was first to pump the fluid to the nozzle; then, the applied voltages were gradually increased until a robust and continuous spinning process emerged under the grounded protection of all the involved parts. These correct procedures are useful for the service life of the instruments and, more importantly, for the safety of the operators and laboratory (Yu et al., 2023a; Yu et al., 2023b; Du et al., 2023). In this investigation, a digital photo of the natural formation of a droplet from the PVP-sucralose solution hung on the nozzle is shown in Figure 5B. With the increase in the applied voltage, the droplet changed its shape from round to “slim” ellipse (Figure 5C, under an applied voltage of 8 kV), to an “elongated” cone shape (Figure 5D, under an applied voltage of 12 kV), and finally to the stable cone shape (Figure 5E, under an applied voltage of 14 kV).

**FIGURE 6:**
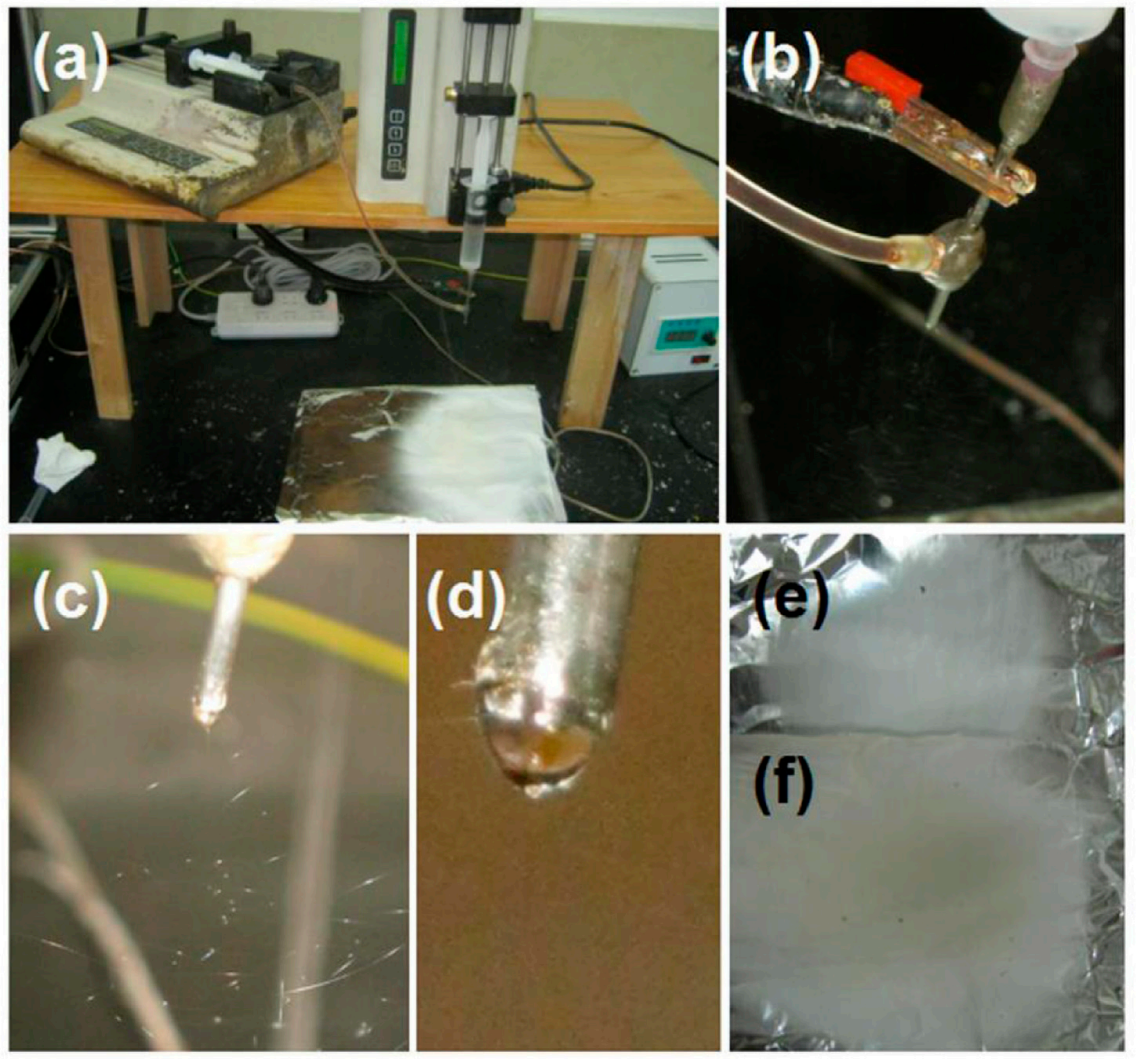
The modified coaxial electrospinning and the resultant nanofibrous mats: **(A)** the preparation of the S5 core–shell nanofibers; **(B)** the convergences of the core fluid, shell fluid, and the electric power on the spinneret; **(C)** the typical working process; **(D)** the compound Taylor cone with the brown core fluid surrounded by the transparent shell solution; **(E)** the resultant S4 nanofibers for comparison; **(F)** the resultant S5 nanofibers.

Based on the investigations of the electrospraying of the core licorice solution (Figure 4) and the single-fluid electrospinning of the shell PVP K90/sucralose blended fluid (Figure 5), the coaxial electrospinning for encapsulating and solidifying the licorice solution was smoothly implemented for fabricating the S5 sample. The series of observations are included in Figure 6. In Figure 6A, it is clear that the S5 collected fibrous mat had a larger surface area than that of the S4 sample, suggesting that the binding and whipping circles were more expanded than for the S4 during the electrostatic repulsion process. The reasons could be that the core water could be retained for a relatively longer time period to maintain the jets in a fluid state for electrical drawing in an extended area. Figure 6B shows the convergences of the core fluid, shell fluid, and the electric power on the spinneret, which were similar to other reports. Under the selected working conditions, the core–shell nanofibers were deposited continuously and robustly. A typical working process is exhibited in Figure 6C. The compound Taylor cone with the brown core fluid surrounded by the transparent shell solution is shown in Figure 6D. Although the commercial licorice solution was encapsulated in the core–shell nanostructures, the S5 collected fibrous mat still showed a slight gray color. In comparison, the S4 fibrous mat from the single-fluid electrospinning of the PVP/sucralose solution showed a complete white color. The digital figures of the S4 and S5 samples are given in Figures 6E, F, respectively.

### 3.2 The morphology and structures of the resultant nanofibers

**FIGURE 7:**
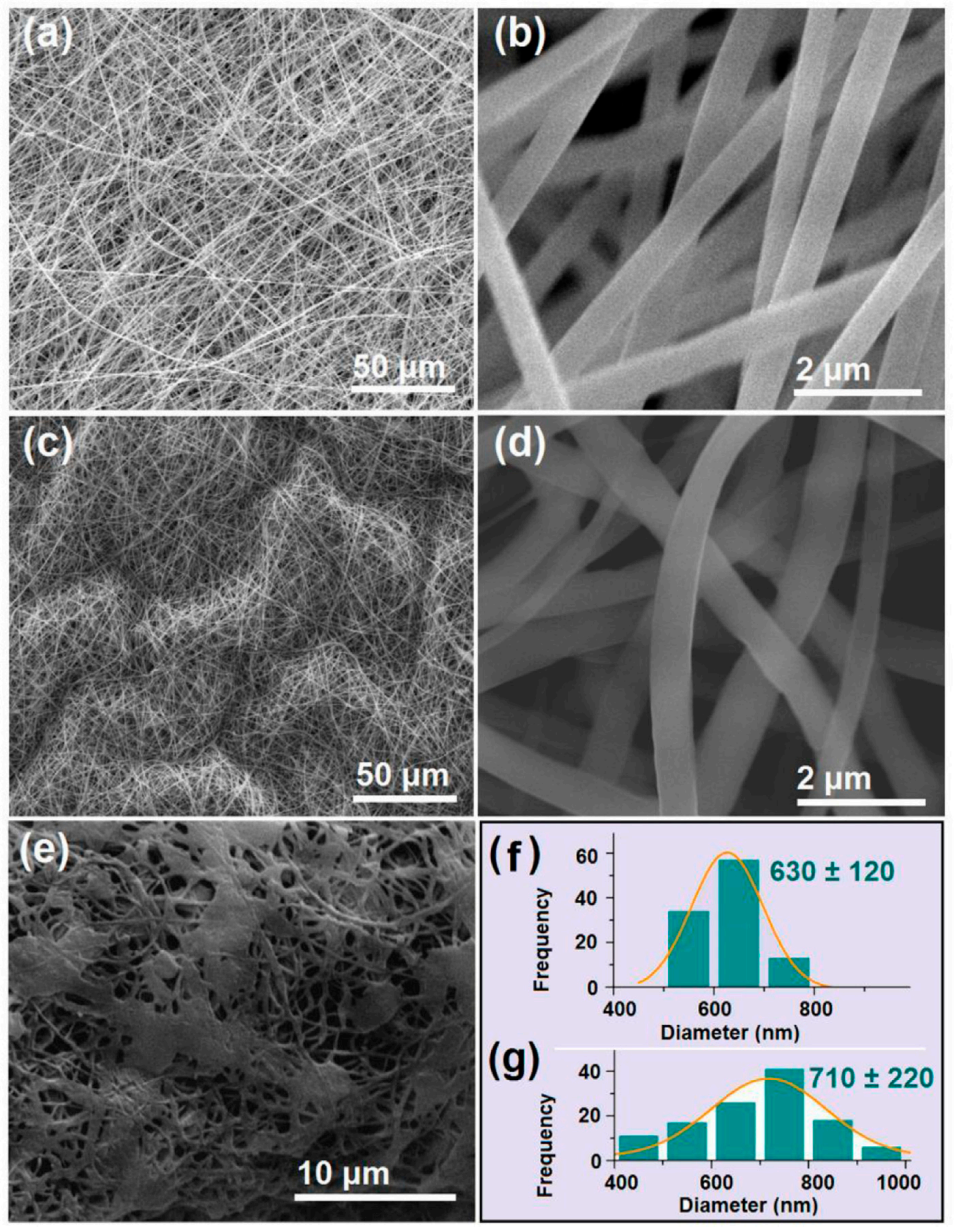
SEM images of the prepared S4 to S6 nanofibers and the diameter distributions of S4 and S5 **(A,B)** SEM images of the S4 monolithic nanofibers at different magnifications **(C,D)** SEM images of the S5 core–shell nanofibers at different magnifications; **(E)** SEM images of the S6 core–shell nanofibers; **(F)** and **(G)** the diameter distributions of the S4 and S5 nanofibers.

The SEM images of the S4 and S5 nanofibers are included in Figure 7. Both the S4 (Figures 7A, B under different magnifications) and S5 (Figures 7C, D under different magnifications) were in straight linear formats without any discerned spindles of beads on them. In contrast, the nanofiber S6 (Figure 7E) exhibited a spoiled morphology, which could be a result of the penetration of the core solution through the shell protection, which sprayed on the collected fibrous mats. The diameter distributions of the S4 and S5 nanofibers are shown in Figures 7F, G, respectively.

Although similar in morphology, the S4 and S5 nanofibers had obvious differences in the deposition state. A comparison of Figure 7B with 7 days indicated that S5 was more crooked than S4. The reasons could be that the unspinnable core licorice solution exerted additional forces to resist the electrical drawing forces along the jet elongation directions and in turn slightly changed the linear formats. Although more solutes passed through the spinneret nozzle in preparing the S5 sample (>0.34 g/h) than for the S4 sample (=0.24 g/h), the average diameter (710 ± 220 nm, Figure 7G) was only slightly larger than the average value of S4 (630 ± 120 nm, Figure 7F). However, the S5 nanofibers had worse uniformity with a broader diameter distribution than the S4 nanofibers. The attendance of the core aqueous solution with the shell electrospinnable PVP/sucralose fluid, on one hand, allowed the compound jets to be drawn for a longer time period, offsetting the increase in the solutes to some extent for the increase in diameter. On the other hand, the simultaneous removing of the core solvents and the shell ethanol made the bending and whipping processes even more complicated, by which the resultant S5 nanofibers showed worse uniformity.

**FIGURE 8:**
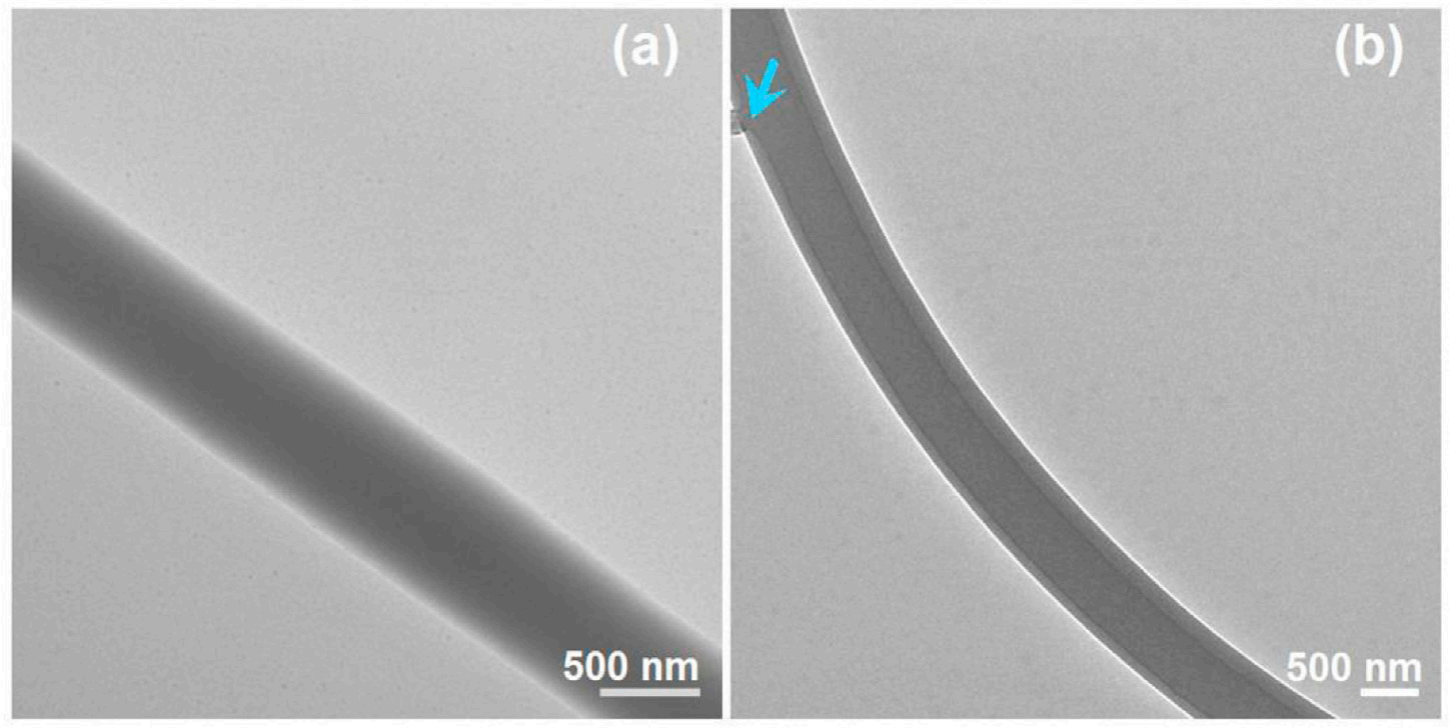
TEM images of the prepared S4 and S5 nanofibers: **(A)** S4; **(B)** S5, the blue arrow indicates a leakage of the unspinnable core liquid.

The TEM images of the prepared S4 and S5 nanofibers are given in Figure 8. As anticipated, the S4 nanofibers had a homogeneous gray level, without any discerned solid-phase separation within the nanofibers. These nanofibers were homogeneous nanocomposites consisting of sucralose and PVP, and the sucralose molecules were distributed all over the PVP matrices in a molecular manner, a propagation of the solution state. In contrast, the S5 nanofibers showed the obvious core–shell structure in Figure 7B. An interesting phenomenon was observed on the evaluated core–shell nanofibers, i.e., an attempted escape was captured occasionally, which is indicated by the blue arrow in Fig b8. Needless to say, the further increase in the core fluid for preparing the S6 nanofibers inevitably resulted in the penetration of more core fluids and a spoiled fibrous mat in Figure 7E.

### 3.3 Physical properties of the S5 nanofibers and the compatibility among the components within the core–shell nanostructures

**FIGURE 9:**
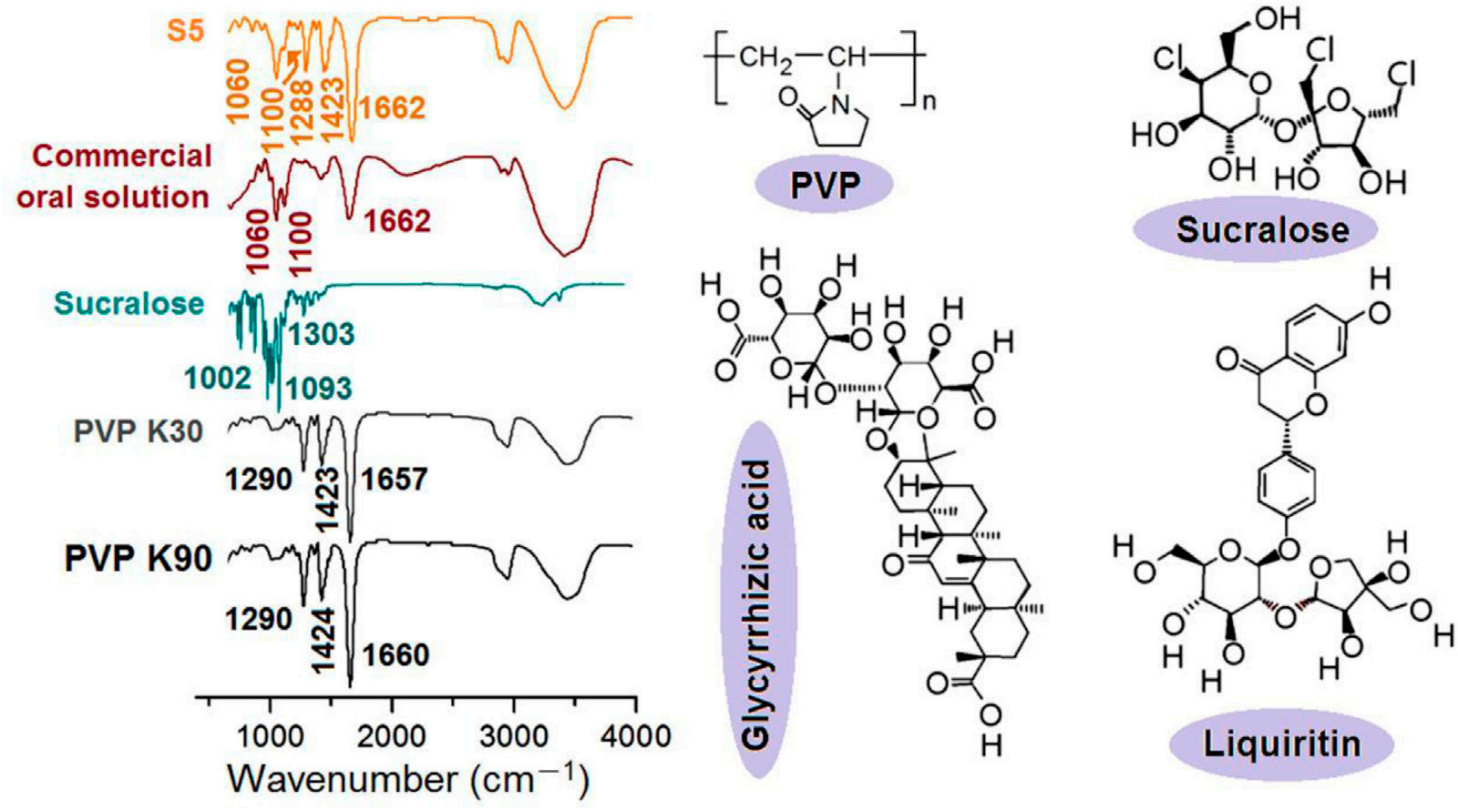
The FTIR spectra of the raw materials and the resultant S5 nanofibers, the molecular formula of PVP and sucralose, and the key elements in the fluidextracts of licorice (i.e., glycyrrhizic acid and liquiritin).

A reasonable selection of a drug carrier must meet several conditions, which at a minimum include good compatibility, manipulation of the drug release behaviors, and good processability (Hameed et al., 2022; Ge et al., 2023; Liu et al., 2023). PVP has a series of products and has a broad application in a wide variety of fields. It has good compatibility with numerous drugs and has been approved for pharmaceutical application by the FDA for many years. However, its applications in herbal medicine are still limited. Thus, the compatibility between the PVP and the licorice fluidextract solution was investigated using FTIR spectra, whose data along with the molecular formula of the raw materials are shown in Figure 9.

Compared with the FTIR spectra of the PVP K30, PVP K90, sucralose, and the commercial oral solution, the S5 electrospun core–shell nanofibers contained characteristic peaks both from the PVP (1662, 1423, and 1288 cm-1), from the commercial oral solution (1060 and 1100 cm^-1^), and also from sucralose (such as 1093 cm^-1^, which should melt to 1100 cm-1). These results suggested the following speculations, which showed the good compatibility between the licorice extracts and the PVP: 1) the commercial oral solution was successfully encapsulated in the PVP nanofibers; 2) no chemical reactions occurred between them, as no new peaks appeared, i.e., a good chemical stability was ensured; 3) the sucralose was completely converted into amorphous nanocomposites, most probably on a molecular scale. These results could also be deduced from the molecular formulas. For example, there are numerous -C=O groups within the molecules of the PVP, whether K90 or K30, and abundant -OH groups present in all other functional molecules (sucralose and glycyrrhizic acid and liquiritin, as the most important active ingredients in licorice); thus, the hydrogen bonds should be broadly present between these molecules for high compatibility and good physical stability. Meanwhile, hydrophobic interactions should also exist between the carbon skeletons of the PVP and the molecules of glycyrrhizic acid and liquiritin from licorice, further enhancing their compatibility.

**FIGURE 10:**
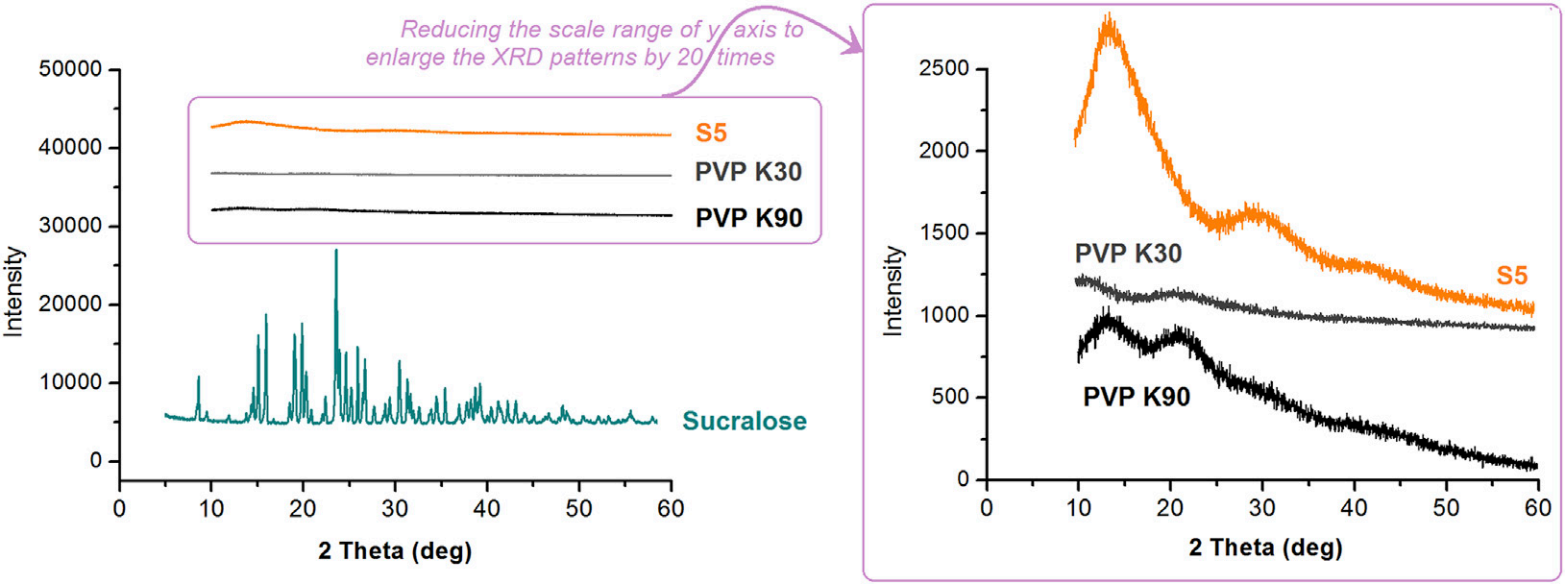
The XRD patterns of the raw materials (PVP K30, PVP K90, and sucralose) and the resultant S5 nanofibers.

In this study, the drug mixture of the commercial oral solution was in a liquid state. However, the active ingredients of glycyrrhizic acid and liquiritin have poorly water-soluble properties. They are soluble in ethanol. When the solution was converted into solid dosage forms, one concern was their physical state within the nanofibers. The XRD patterns of the raw materials (PVP K30, PVP K90, and sucralose) and the resultant S5 nanofibers are exhibited in Figure 10. Apparently, the S5 fibrous mats were amorphous composite materials. Not only was the crystalline sucralose presented in an amorphous state, losing its original powder crystalline state, but all the active ingredients solidified in the nanofibers from the licorice fluidextracts formed an amorphous state with the polymeric matrices, which was indicated by the TEM and FTIR results.

### 3.4 Functional fast dissolution performance

Compared to the solid dosage forms, the liquid dosage forms have the advantages of simple administration and often a fast dissolution and delivery process after oral administration. However, their disadvantages are also obvious, i.e., poor stability and difficulty in terms of encapsulation and shipping. Provided the solid dosage forms can be dissolved all at once, they should hold the advantages of both the solid dosage forms and the liquid dosage forms. Thus, three different methods were developed to assess the functional performance of the fast dissolution of the resulting S5 core–shell nanofibers.

**FIGURE 11:**
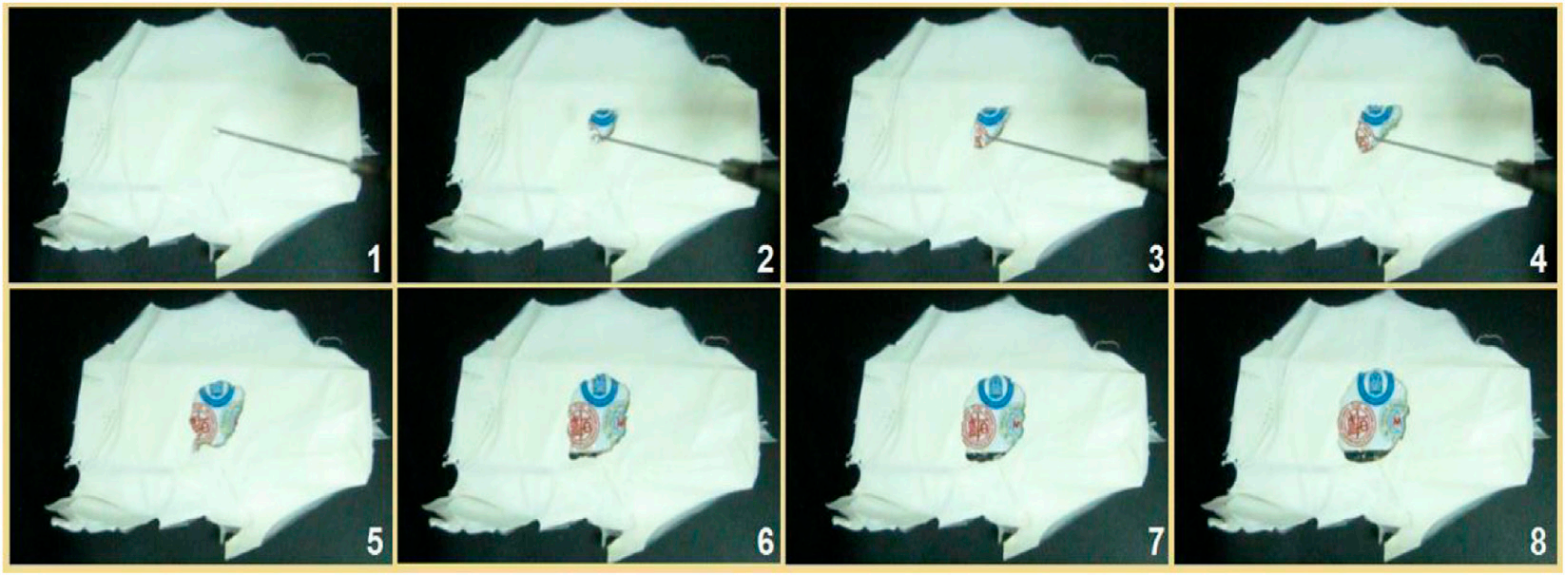
The fast dissolution when a drop of water was placed on the collected core–shell nanofibers on a glass slide, under which was an image of the three logos of the first to third authors’ high schools. The time span from image 1 to image 8 was 5 seconds.

Shown in Figure 11 are the results of the fast dissolution when a drop of water was placed on the collected core–shell fibrous mats, which were collected on a glass slide. When contacted by the water, the fibrous mats were dissolved, and the circle expanded very quickly, as indicated by the gradual appearance of the underlying image of three logos of the first to the third authors’ high schools. The time span from image 1 to image 8 was 5 s.

**FIGURE 12:**
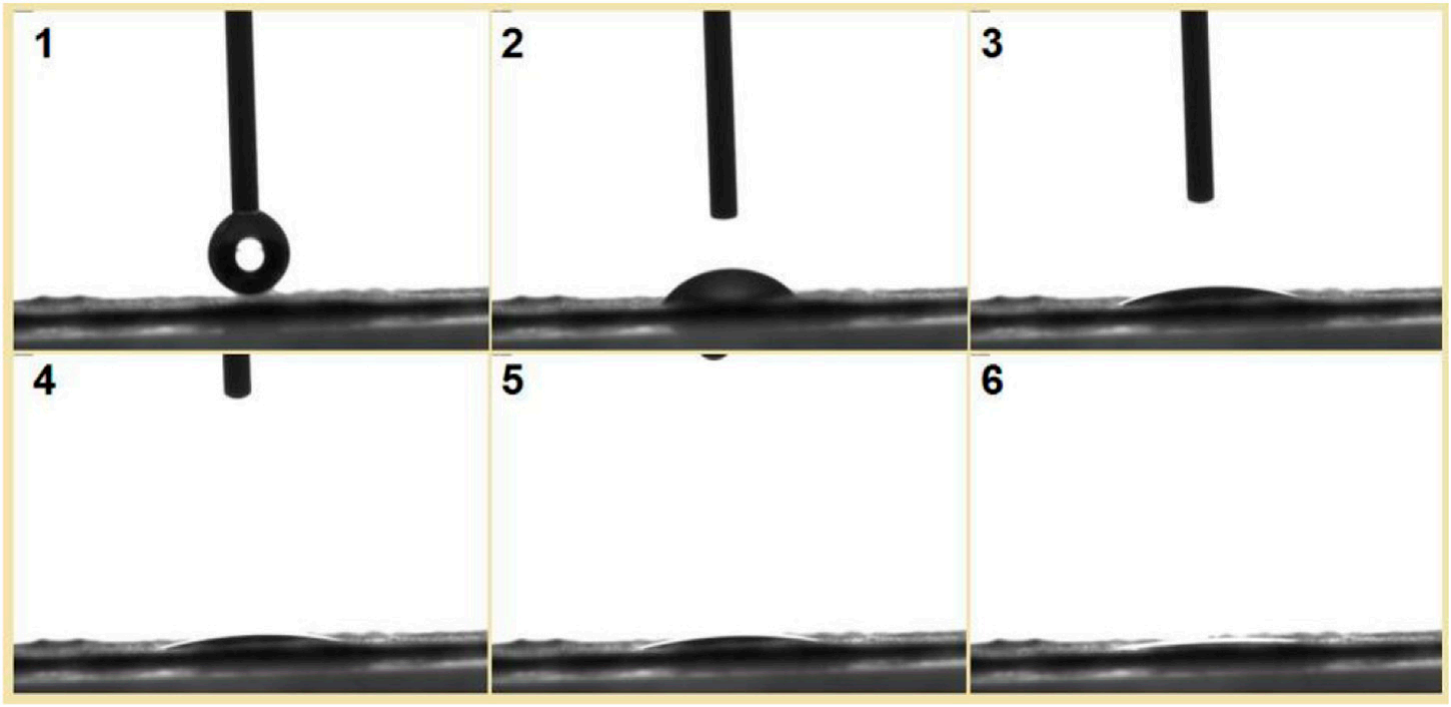
A drop of water with a volume of 3 μL was placed on the collected nanofibers, and the disappearance process was recorded using a water contact angle measurement instrument. The time span from image 1 to image 6 was 3 s.

To be further observe the fast dissolution process of the S5 nanofibers, a water contact angle instrument was used to record the processes. Shown in Figure 12 is a water droplet retreating process. The drop of water had a volume of 3 μL; its disappearance from image 1 to image 6 in Figure 12 took 3 s.

**FIGURE 13:**
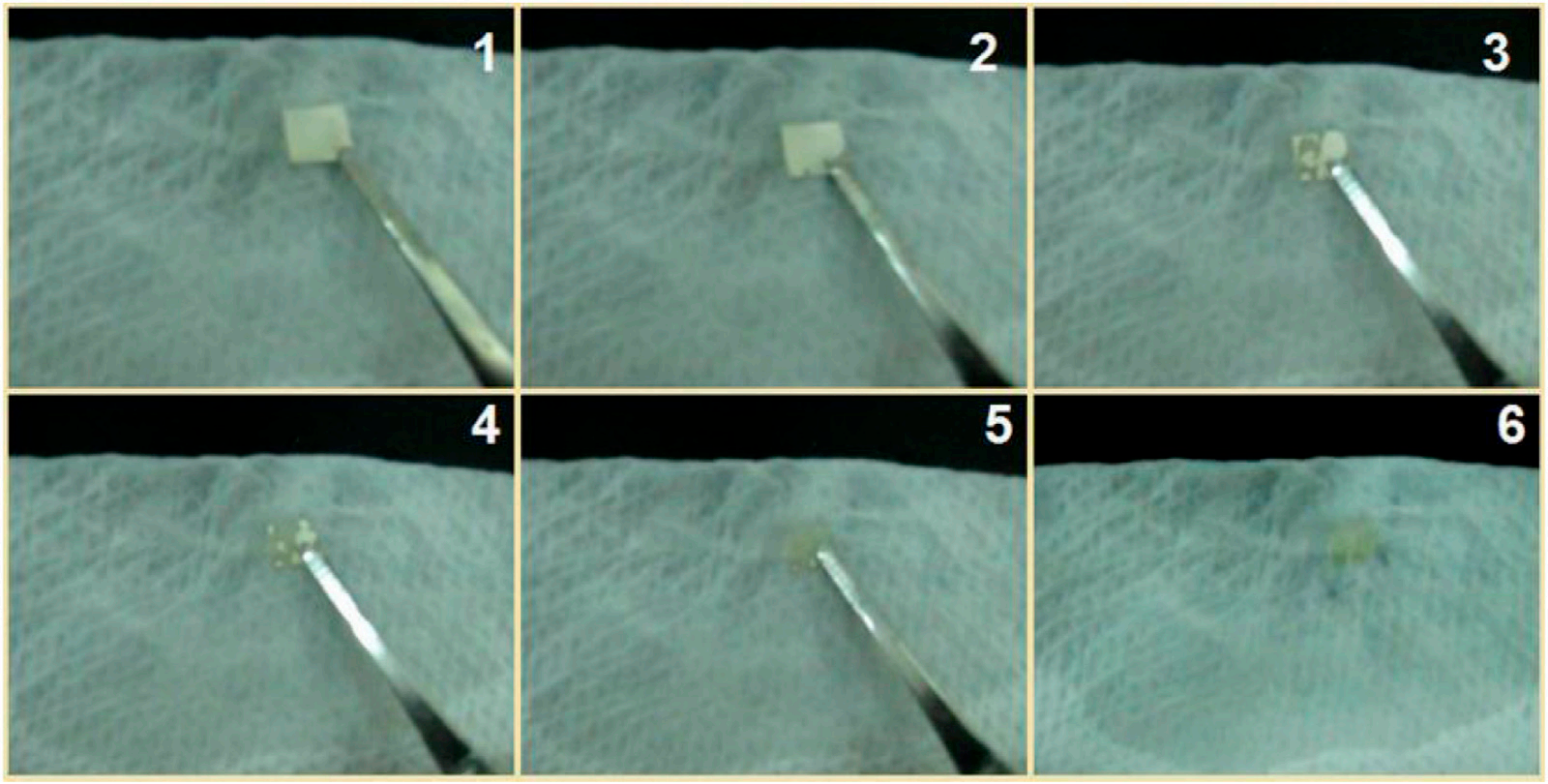
A piece of fibrous mat was placed on a sheet of wet paper on the laboratory desk; the whole dissolution process took 2 s from image 1 to image 6.

In traditional pharmaceutical methods, there are several different kinds of solid dosage forms, such as fast disintegration tablets, buccal films, sublingual tablets, and mini tablets. These dosage forms are very useful for oral administration to the elderly, children, people with dysphagia, and persons in the case of water shortage. When they are placed in the mouth, the limited moisture in the oral fluid is enough for dissolving the loaded drugs. Thus, an additional experiment was designed to mimic this case, in which the fibrous S5 mat was placed on a sheet of wet paper (a tongue analogue) on the laboratory desk. The fast disappearance processes recorded by a camera are shown in Figure 13. The time from the white square in image 1 to the blur brown residue in image 6 was 2 s. This indicates that the S5 electrospun nanofibers are potential candidates for fast delivery of licorice’s active ingredients through oral administration.

The abovementioned three methods showed that there was no problem with the fast dissolution of the S5 electrospun fibrous mats, i.e., they had the advantages of the commercial compound solution for simple oral administration. Meanwhile, the active ingredients should be stabler when they are sealed into the core sections of the core–shell nanofibers and present in a solid state, which will be further investigated. In addition, the ethanol in the licorice fluidextract commercial solution was totally removed by the electrospinning procedure; thus, the electrospun S5 mats should be safer. Any residue of ethanol in the S4 and S5 samples and its contents in the commercial solution will be detected in the near future. Thus, compared with the solution, on one hand, the S5 electrospun fibers are more convenient for encapsulation and shipping and are more stable for long-term storage. On the other hand, they can ensure a safer and more convenient oral administration (the commercial licorice solution needs a special vessel to measure a certain volume liquid for oral administration).

### 3.5 Mechanism for the rapid dissolution process

**FIGURE 14:**
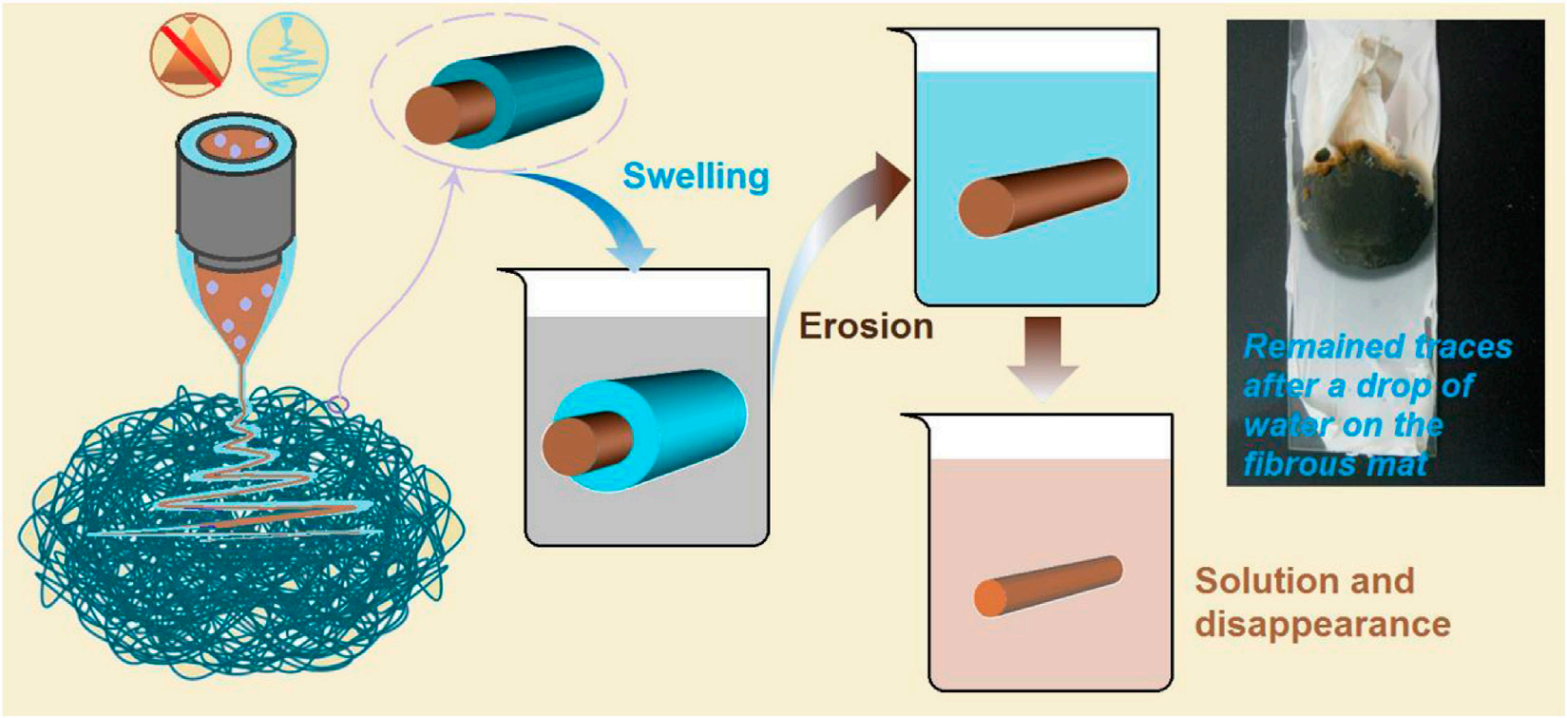
The mechanisms of the rapid dissolution of the S5 nanofibers as candidates for oral fast disintegrating films. The top-right inset is a digital photo of the remaining traces, which were formed after the drop of water on the fibrous mat naturally dried.

Based on the above investigations, the fast conversion of the S5 electrospun core–shell nanofibers to a liquid state to deliver the contained active ingredients can be ensured. In addition to maintaining the advantages of liquid dosage forms, the electrospun core–shell nanofibers provide other benefits, including simple aftertreatment for commercial applications, convenient administration, and enhanced stability. Meanwhile, the mechanism for the rapid dissolution processes is clear, as diagrammed in Figure 14.

Three key elements played important roles in the materials’ conversions and applications. Firstly, the PVP K90, as a shell polymeric matrix, was soluble in water and had a good electrospinnability, by which the unspinnable core licorice solution was effectively solidified into the S5 core–shell nanofibers. During the dissolution processes, the PVP K90 shell contacted the water first. It experienced a typical dissolution process of the macromolecules, i.e., absorbing water, swelling, disintegration, and dissolving into the bulk solution. Secondly, we used the PVP K30 as an additive in the commercial oral solution, which increased the viscosity of the core fluid to ensure a successful coaxial working process during preparation. During storage, PVP K30 can effectively keep the active ingredients in an amorphous state, preventing the possible recrystallization. When the fibrous S5 were utilized for oral administration, the PVP K30 quickly absorbed the water and released the loaded active ingredients in an erosion manner, quickly. The third element in the core–shell structure was the intentional arrangements of the components within it. If the shell and core section were to exchange places, the resultant core–shell nanofibers might still show fast dissolution; however, the stability might be reduced because the active ingredients would be located in the shell section; meanwhile, the PVP K30 has a stronger hygroscopicity than PVP K90. The proposed mechanism was demonstrated by a simple experiment. Shown in the top-right inset of Figure 14 is a digital photo of the remaining traces, which were formed after a drop of water was placed on the fibrous mat and later naturally dried. The dissolved active ingredients from the core sections are the brown color within the boundaries of the circle.

With the gradual expansion of electrospinning in pharmaceutics, its ability to convert dosage forms is drawing increasing attention (Németh et al., 2022; Shokoya et al., 2023). The well-known unique properties of electrospun nanofibers (including small diameter, high porosity, large surface area, and simple fabrication) are showing merit for pharmaceutical applications, such as the amorphous state of drugs, their simple storage and good stability, fast dissolution, simple encapsulation of all kinds of active ingredients (oil, herbal medicines, proteins and peptides, and small chemical molecules), strong capability of synthesizing structural hybrids from a combination of filament-forming polymeric excipients, as well as excipients without electrospinnability (such as lipids, surfactants, and even inorganic materials), and the simple conversions of electrospun nanofibers as intermediate dosage forms to final medical products (Weaver et al., 2022). Understanding the mechanism of action is conducive to building new drug delivery systems and upgrading traditional dosage forms.

## 4 Conclusion

In this study, modified coaxial electrospinning was developed through a combination of a spinnable blended solution containing PVP K90 and sucralose as a shell fluid and an unspinnable commercial oral licorice solution as a core fluid. Some PVP K30 was added to the commercial oral solution. Its EHDA-treating properties were investigated to convert it into solid core–shell nanofibers through the coaxial working process. The SEM and TEM assessments verified that the resultant nanofibers had a linear morphology, and the designed core–shell nanostructures encapsulated the active ingredients in the commercial oral solution. The FTIR spectra and XRD patterns indicated that the active ingredients of licorice (such as glycyrrhizic acid and liquiritin) had good compatibility with the polymeric matrix PVP K30, and they were kept in an amorphous state in the nanofibers. Three different types of methods (including water dripped on the fibrous mats and fibrous mats placed on a surface wet with water) were developed, and the fast dissolution properties of the electrospun core–shell nanofibers were demonstrated. A mechanism for a fast dissolution process was proposed. The present study pioneered a proof-of-concept demonstration of a method by which traditional liquid dosage forms can be upgraded into solid dosage forms and retain the drug delivery advantages of both the traditional liquid dosage forms and the solid dosage forms. Based on the reported protocols, there would be more dosage conversion possibilities for the safer and more convenient drug delivery through core–shell nanofibers *via* coaxial electrospinning. Certainly, some other investigations such as the residue ethanol in the core-shell hybrids, the stability and the toxicity assessments should be further conducted for real commercial applications.

## Data Availability

The original contributions presented in the study are included in the article/Supplementary Material, further inquiries can be directed to the corresponding authors.
