# Corrigendum: Fast and convenient delivery of fluidextracts liquorice through electrospun core-shell nanohybrids

**DOI:** 10.3389/fbioe.2025.1596937

**Published:** 2025-06-27

**Authors:** Hang Liu, Yelin Dai, Jia Li, Ping Liu, Wenhui Zhou, Deng-Guang Yu, Ruiliang Ge

**Affiliations:** ^1^ School of Materials and Chemistry, University of Shanghai for Science and Technology, Shanghai, China; ^2^ Wenqi Middle School, Shanghai, China; ^3^ Qingpu Campus, High School Affiliated to Fudan University, Shanghai, China; ^4^ Institute of Infectious Disease and Biosecurity, School of Public Health, Fudan University, Shanghai, China; ^5^ The Base of Achievement Transformation, Shidong Hospital Affiliated to University of Shanghai for Science and Technology, Shanghai, China; ^6^ Institute of Orthopaedic Basic and Clinical Transformation, University of Shanghai for Science and Technology, Shanghai, China; ^7^ Department of Outpatient, The Third Affiliated Hospital, Naval Medical University, Shanghai, China

**Keywords:** coaxial electrospinning, core–shell structure, liquorice, solidification, dosage forms, process–structure–performance relationship

In the published article, there was an error in Figure 10 as published. The XRD patterns of PVP K30 and PVP K90 may mislead the readers. Thus, the two samples were re-prepared and re- tested. The corrected Figure 10 and its caption “Figure 10 The XRD patterns of the raw materials (PVP K30, PVP K90, and sucralose) and the resultant S5 nanofibers.” appear below.

**FIGURE 10:**
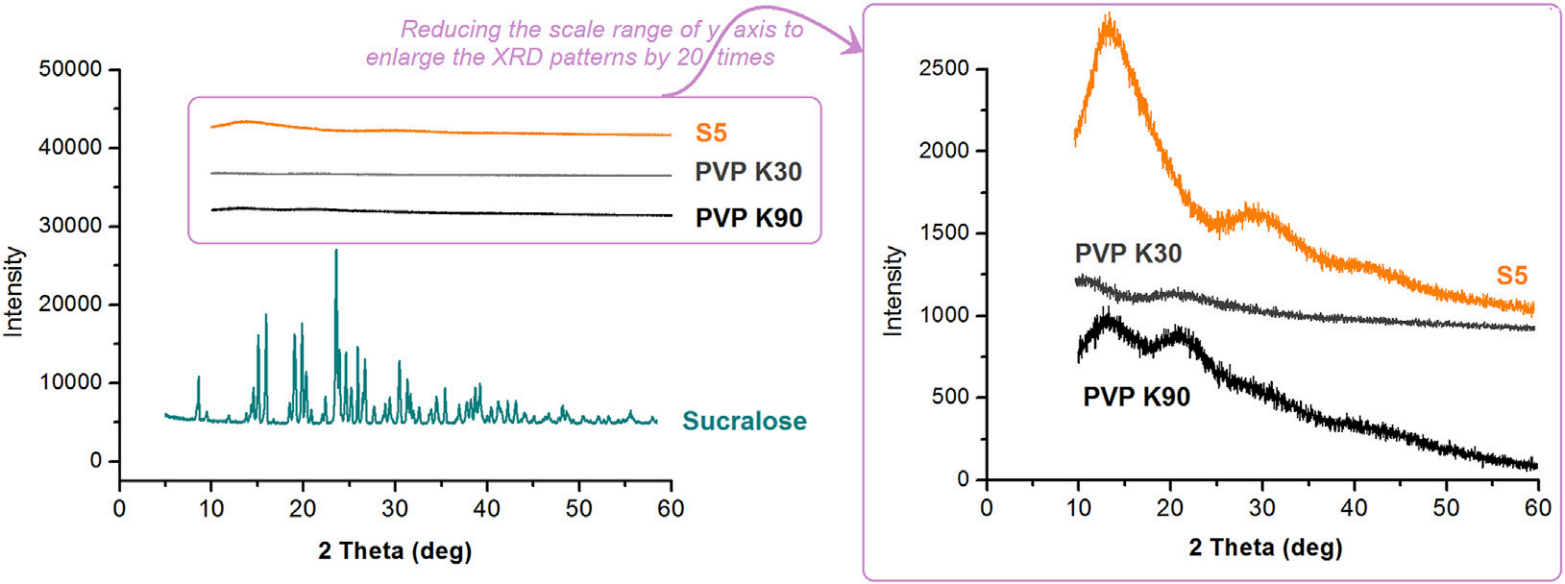
The XRD patterns of the raw materials (PVP K30, PVP K90, and sucralose) and the resultant S5 nanofibers.

The authors apologize for this error and state that this does not change the scientific conclusions of the article in any way. The original article has been updated.

